# Current Trends in Messenger RNA Technology for Cancer Therapeutics

**DOI:** 10.34133/bmr.0178

**Published:** 2025-04-09

**Authors:** Ali Afzal, Muddasir Hassan Abbasi, Shaaf Ahmad, Nadeem Sheikh, Muhammad Babar Khawar

**Affiliations:** ^1^ Applied Molecular Biology and Biomedicine Lab, Department of Zoology, University of Narowal, Narowal, Pakistan.; ^2^ Department of Zoology, University of Okara, Okara, Pakistan.; ^3^ King Edward Medical University/Mayo Hospital, Lahore, Punjab 54000, Pakistan.; ^4^ Cell & Molecular Biology Lab, Institute of Zoology, University of the Punjab, Lahore, Pakistan.

## Abstract

Messenger RNA (mRNA)-based therapy has revolutionized cancer research by enabling versatile delivery systems for therapeutic applications. The future of mRNA-based cancer therapies shows promise amidst challenges such as delivery efficiency, immunogenicity, and tumor heterogeneity. Recent progress has adapted various strategies such as design flexibility, scalable production, and targeted delivery capabilities to enhance the potential in personalized cancer therapy. Further research to optimize delivery for enhanced outcomes and efficacy in solid tumors is warranted. Therefore, we aim to explore the current landscape and future prospects of mRNA technology across various therapeutic platforms.

## Introduction

Cancer remains a leading global health challenge, ranking as the second most common cause of mortality worldwide [1]. Strikingly, The Global Burden of Disease reveals that cancer ranks second only to cardiovascular diseases concerning mortality, disability-adjusted life years, and years of life lost globally [2]. Advances in cancer therapy have reduced mortality rates considerably, which averts an estimated 3.8 million deaths [3]. The supplementary statistics around the globe for 2022 are summarized in Fig. S1. Moreover, the American Cancer Society in 2024 predicts that there will be 2 million new cases of cancer and 0.6 million mortality rate due to cancer in the United States; nonetheless, the death rate seemed to drop through 2021, which results in an overall reduction of 33% of cases since 1991 [4].

Despite progress, challenges such as delivery inefficiency, tumor heterogeneity, and off-target effects persist and more focused targeted therapies are warranted. Progressively, there has been a major shift toward therapies designed to specifically target tumors, thus minimizing harm to surrounding healthy tissues. Remarkably, the success in messenger RNA (mRNA)-based therapeutics has garnered widespread attention and achievement in the context of COVID-19 mRNA vaccines, currently available commercially, for instance, Pfizer-BioNTech and Moderna. The adaptability and rapid deployment of mRNA platforms underscore their potential, thereby extending beyond infectious diseases to encompass applications in cancer therapy.

Currently, mRNA therapeutics offer various benefits over conventional approaches. One of the main benefits is transient transfection without the need for genomic integration, which is an additional avenue of discussion. Consequently, in vitro transcription of mRNA of various types as shown in Table 1 have shown great promise via enabling the cells to synthesize mRNA-encoded proteins without interrupting or editing the genomic DNA [5]. Secondly, considerable efforts have been deployed to enhance the stability with lesser immunogenicity in vitro and in vivo by employing nucleoside modification [6], purification, and improved sequencing [7]. To further improve the stability, a pivotal-efficacy trial (NCT04368728) using a nonviral delivery via lipid nanoparticle (LNP) formulation (BNT162b2) was utilized with nucleoside-modified RNA to encode spike protein [8], thus conferring 95% efficacy against coronavirus. However, various other mRNA LNP-formulated vaccines targeting cancer-associated neoantigens are being investigated in phase I/II trials [9]. Thirdly, mRNA-based therapeutics offer efficient translation of encoded proteins either to regulate tumor microenvironment (TME) or to activate immune responses. For example, p53, a tumor suppressor, was restored using mRNA-nanoparticle (NP) delivery, thereby improving the tumor sensitivity to rapamycin inhibitors [10], which shows the promise of mRNA-encoded tumor suppressors in tumor inhibition. Additionally, mRNA-encoded cytokines activate immune responses against tumor in vivo either in targeted lesions or in remote TME sites, which include induction of interferon-γ (IFN-γ), increased infiltration of B +ve T lymphocytes, and developing immune memory [11]. Last but not least, higher transfection rates have been observed in cancer immunotherapy while in vitro encoding of chimeric antigen receptor (CAR) mRNA in lieu of antigen/viral recognition in animal models of leukemia, prostate, and hepatic cancer [12].

**Table 1.** Summary of initial breakthroughs in mRNA research

Study	Year	mRNA specifics	Methodology	Contribution in mRNA research	References
Dimitriadis et al.	1978	mRNA encapsulation in liposomes protects from degradation.	Injection of mRNA into mouse spleen lymphocytes; use of liposomes for delivery.	Demonstrated mRNA translation in differentiated cells using mRNA–liposome complexes.	[147]
Malone et al.	1989	RNA transfection using cationic lipids; role of mRNA capping on translation.	Transfection of luciferase mRNA into cells using lipofectin.	Developed efficient RNA transfection method; studied mRNA translation factors and efficiency.	[148]
Wolff et al.	1990	mRNA and DNA vectors for protein expression injected into mouse muscle.	In vivo injection into mouse skeletal muscle.	Demonstrated protein expression from injected mRNA in vivo, comparable to in vitro transfection.	[149]
Karikó et al.	2005	RNA activates TLRs; modified nucleosides reduce dendritic cell activation.	Experiments with dendritic cells exposed to modified RNA.	Identified immunomodulatory effects of RNA modifications; implications for mRNA therapeutics.	[6]
Karikó et al.	2008	Pseudouridine-modified mRNA enhances translation and reduces immunogenicity.	In vitro and in vivo testing of pseudouridine-modified mRNA in mice.	Showed enhanced stability, translation efficiency, and reduced immunogenicity of modified mRNA.	[150]
Sahin et al.	2017	RNA-based poly-neo-epitope vaccine induces T-cell responses against cancer.	Application in melanoma patients; assessment of T-cell responses.	Demonstrated personalized cancer immunotherapy using RNA-based vaccines targeting neo-epitopes.	[151]
Polack et al.	2020	BNT162b2 mRNA vaccine encoding SARS-CoV-2 spike protein.	Large-scale placebo-controlled trial for COVID-19 vaccine.	Developed and tested mRNA vaccine against SARS-CoV-2; demonstrated high efficacy and safety.	[8]

**Fig. 1.**
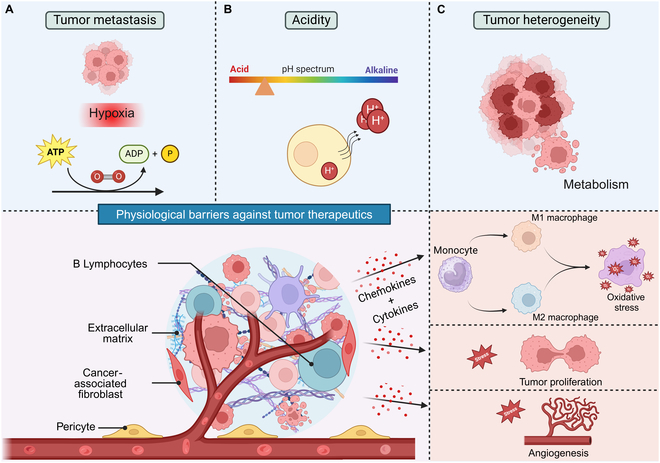
Mechanistic depiction of tumor microenvironment (TME) barriers. Several physiological factors including immune cells, extracellular matrix, hypoxia, and acidity affect the physiology of TME in a number of ways. (A) Tumor-immune interactions and oxidative stress, on the other hand, have a major role in influencing the TME. All of these factors enhance tumor metastasis, (B) acidity, and (C) heterogeneity, which are the potential barriers for mRNA cancer therapy.

However, the TME poses some key barriers to therapeutic efficacy, including immunosuppressive cells, hypoxia, and dense extracellular matrix (Fig. 1). Overcoming these challenges is critical for advancing mRNA-based cancer therapies. Density of extracellular matrix is one of the barriers of TME. For instance, in cartilage, the extracellular matrix spacing is approximately 20 nm, while conventional NPs often exceed 50 to 60 nm in diameter, thereby resulting in poor penetration [13]. Apart from this, antibodies face reduced efficacy due to upregulated immune checkpoints like programmed death-ligand 1 (PD-L1) [14], and costimulatory agents struggle with T-cell exhaustion from chronic antigen exposure [15]. Cytokines often induce off-target inflammation or are neutralized by suppressive factors such as transforming growth factor-β (TGF-β) [16]. Protein replacement therapies are compromised by protease-rich conditions [17,18], and adoptive cell therapies (e.g., CAR T/NK) are inhibited by immunosuppressive cells [19]. Even CRISPR-Cas9 systems face delivery inefficiencies in fibrotic and heterogeneous tumors [20]. Addressing these barriers requires innovative strategies, such as mRNA-based platforms, which offer modular and adaptable solutions to overcome TME-driven limitations.

These promises in mRNA therapeutics bring us to the crux of our exploration—the capability of mRNA delivery platforms in revolutionizing cancer treatment. The precision of mRNA delivery systems allows for the targeted administration of therapeutic payloads, offering a promising avenue for personalized medicine in the cancer therapeutics. Therefore, despite these advancements, there is an imperative need for dedicated exploration to optimize delivery systems for mRNA payloads. In the next sections, (a) we aim to explore the versatility of mRNA delivery methods and (b) their applications in various therapeutic areas, including vaccines, antibodies, costimulatory agents, cytokines, protein replacement therapy, adoptive cell therapy, and gene editing with an emphasis on (c) potential future prospects of mRNA-based therapies.

## Versatile Delivery of mRNA

Owing to its unique attributes, such as ease of manipulation in design, scalability for industrial production, and versatility, mRNA—as a potential therapeutic option—has garnered attention [21]. Furthermore, achieving high cell transfection rates through nanodots [22] and the capability to achieve organ selectivity via multidimensional screening and isomeric elucidation [23] highlight its capacity to deliver mRNA in vivo. This platform holds promise in surmounting various barriers, either systemic or cytoplasmic, thereby improving translation of tissue- or cell-specific proteins. Lipid-based nanocarriers have been instrumental in improving mRNA transfection into vehicle cells by enhancing its stability and enabling an efficient uptake and cytoplasmic transport [24]. Among them, cationic or ionizable LNPs have been well studied as materials for mRNA delivery (discussed in detail in later sections).

An innovative synthesis strategy, the microfluidic mixer approach, offers numerous advantages, including large-scale production of LNPs efficient for encapsulation and uniformity, thus streamlining the manufacturing of mRNA products adhering to the standards of good manufacturing practices [25]. Enhancing the transfection efficiency of mRNA-based gene therapy is achievable through NP-based delivery systems, such as poly(amidoamine)-based NPs, which have demonstrated promising outcomes in delivering mRNA to targeted cells and tissues [26]. However, challenges persist, notably in achieving efficient escape from endosomes and precise targeting of tissues/cells, posing exhaustive hurdles for mRNA-based therapies [27]. Key factors for the successful expansion of mRNA technology in treating genetic diseases like cancer include developing clinically applicable mRNA transporters, ensuring stability in systemic circulation, and mitigating immunogenicity [28]. These advancements lay the groundwork for further exploration and development of mRNA-based therapies with enhanced efficacy and specificity for future biomedical applications.

## mRNA-Based Vaccines

**Fig. 2.**
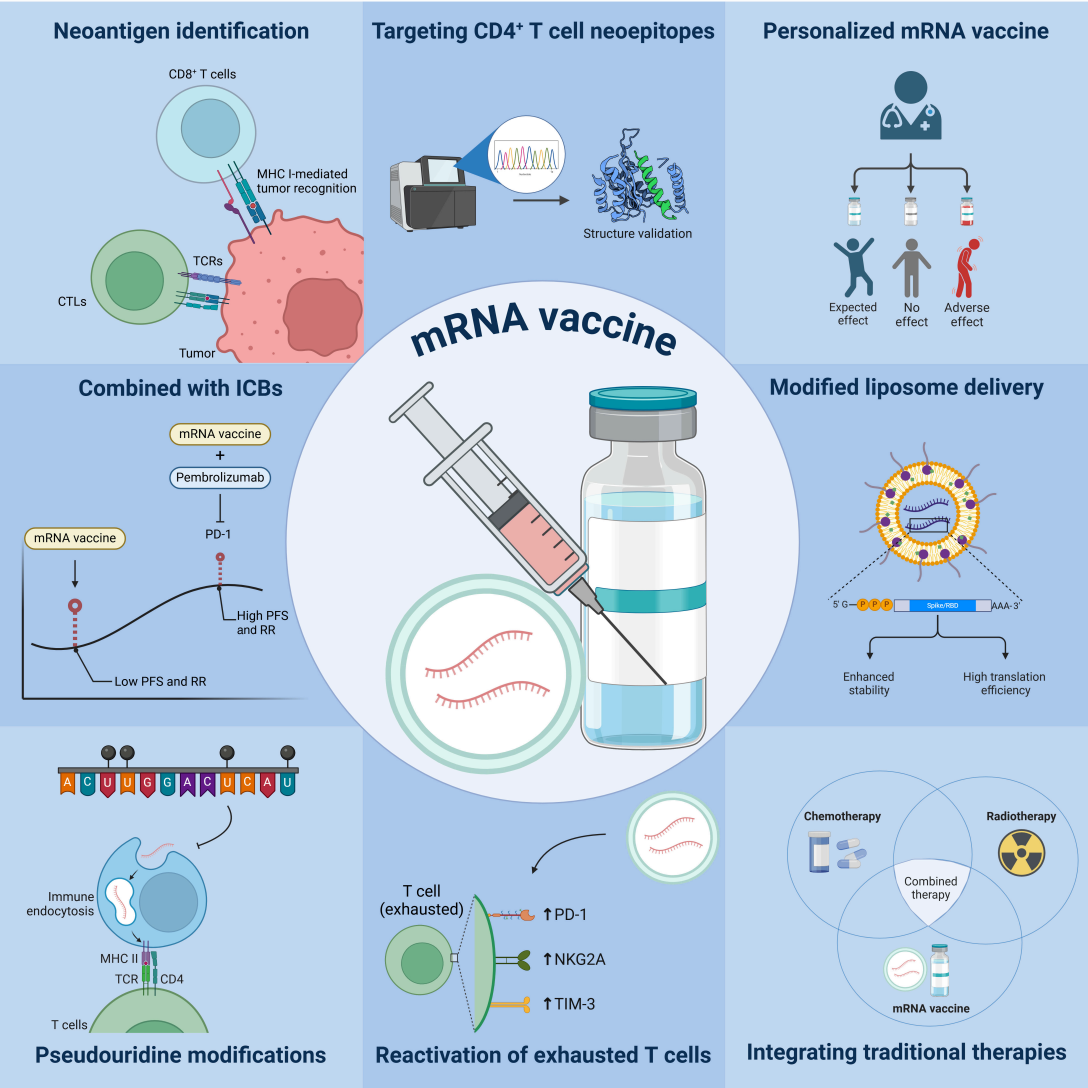
Mechanistic innovations in mRNA-based vaccines. Enhanced neoantigen identification is achieved through advanced algorithms that predict immunogenic neoepitopes recognized by CD8^+^ T cells via the MHC I pathway. Targeting CD4^+^ T cell neoepitopes is enhanced by emerging algorithms. Personalized mRNA vaccine platforms tailor vaccines to tumor antigens of patients with increased efficacy. Combination therapy with immune checkpoint inhibitors, such as pembrolizumab, shows synergistic effects, which reprograms the TME for a stronger immune response. Modified liposome delivery systems enhance mRNA stability and translation efficiency to improve immunocyte uptake. Pseudouridine modifications in mRNA sequences increase stability and protein production, which reduces immune recognition of the mRNA as foreign invader. Reactivation of exhausted T cells by mRNA vaccines restores their function to enhance anticancer capabilities. Finally, integrating mRNA vaccines with traditional treatments, like chemotherapy and radiation, leverages the strengths of both to provide a multifaceted attack on cancer cells and improve patient outcomes.

mRNA cancer vaccines are considered safer and effective due to their superior safety profile compared to other vaccine types like pDNA and viral vectors. They are easy to produce, rapidly developed, and have minimal genetic elements required for protein expression. mRNA vaccines can deliver various therapeutic agents, modulate the immune response, and generate cancer-specific T cells, making them versatile and promising for cancer immunotherapy (Fig. 2) [29]. The first Food and Drug Administration (FDA)-approved cancer vaccine against hormone-refractory prostate cancer is sipuleucel-T (Provenge) [30]. Currently, 2 vaccinations have been licensed to prevent hepatocellular carcinoma (HCC) caused by the HBV, while 70% of cervical cancer is caused by the human papillomavirus (HPV) [31]. These early successes served as motivation for developing vaccines against antigens specifically expressed by tumor cells referred to as tumor-associated antigens (TAAs) [32]. Many of these vaccines are employed either on their own or in combination with secondary treatment modalities, for instance, cytokines and checkpoint blockade inhibitors [33].

Intriguingly, neoantigens have been of great interest in the field of mRNA vaccines. These are tumor-specific proteins arising from somatic mutations that are absent in normal tissues. Unlike self-antigens, neoantigens are recognized as foreign by the immune system [34], making them ideal targets for cancer immunotherapy. mRNA-based vaccines leverage this concept by encoding tumor-specific neoantigens, stimulating a strong and precise immune response [35]. By priming T cells against these unique antigens, mRNA vaccines enhance antitumor immunity while minimizing off-target effects [36]. The use of neoantigen-based mRNA vaccines has shown promise in recent clinical trials (as summarized in Table 2), demonstrating improved recurrence-free survival (RFS) in melanoma and pancreatic cancer patients. Introducing this concept early provides a foundation for understanding the mechanism and potential of mRNA-based cancer vaccines.

**Table 2.** Summary of trials utilizing mRNA-based therapies in cancer treatment

mRNA therapeutic	Phase	Indication/disease	Key findings	Status	Failures/negative outcomes	Trial ID
BNT162b2, mRNA-1273	Phase 3	Immunocompromised individuals with multi-disease conditions (including lymphoid malignancies)	Third dose improved serological and T-cell responses in the majority of patients; 90% of low responders showed increased antibodies.	Completed	54% of baseline nonresponders showed no response after the third dose.	ISRCTN 15354495
mRNA COVID-19 vaccine	Phase 2	Cancer patients, including those with hematologic malignancies	Third dose led to seroconversion in 57% of previously seronegative patients; fourth dose boosted immunity in 67% of poor responders; durable immunity observed at 6 months.	Completed	Limited neutralization against Omicron variant; inadequate response linked to low IgM and CD19 counts.	NCT05016622
BNT162b2	Phase 3	Participants with past or active neoplasms (malignant or benign/unknown)	Vaccine efficacy was 94.4% after up to 6 months post-dose 2, comparable to the overall population; safety profile consistent with general trial population.	Completed	Higher adverse event (AE) incidence in the vaccine group (95.4 vs. 48.3 per 100 person-years); 3 withdrawals due to vaccine-related AEs.	NCT04368728
BNT162b2	Not specified	Multiple myeloma (MM) and myeloproliferative malignancies (MPM)	Immunogenic response observed: 88% in MPM patients, lower in MM patients (78.6%); MM patients on anti-CD38 therapy had considerably reduced response.	Completed	Reduced response in MM patients, especially those on anti-CD38-based treatments; MM patients advised to maintain preventive measures post-vaccination.	Prot. N-1463/21
Autogene Cevumeran	Phase I	Pancreatic ductal adenocarcinoma (PDAC)	Induced neoantigen-specific T cells in 8/16 patients; responders had longer recurrence-free survival.	Active, not recruiting	8/16 patients showed no T-cell expansion; recurrence in nonresponders at 13.4 months	NCT04161755
Neoantigen mRNA vaccine	Phase I/II	Metastatic gastrointestinal cancer	Safe, induced mutation-specific T-cell responses	Completed	No objective clinical responses in 4 patients	NCT03480152
Protamine-stabilized mRNA (Melan-A, Tyrosinase, gp100, Mage-A1, Mage-A3, and Survivin)	Not specified	Metastatic melanoma	Feasible, safe, reduced immunosuppressive cells, increased vaccine-directed T cells	Completed	Limited clinical response (1/7 patients showed complete response)	NCT00204607
CV9202 (self-adjuvanting mRNA targeting NY-ESO-1, MAGEC1, MAGEC2, 5T4, Survivin, MUC1)	Phase 1	Advanced non-small cell lung cancer (NSCLC)	Feasible, combined with radiotherapy, aims to induce immune responses	Ongoing	None reported yet; primary endpoint: Grade >3 adverse events	NCT01915524
Pfizer/BioNTech BNT162b2 Wildtype/Omicron BA.1 and BA.4/BA.5	Phase 2	COVID-19 (vaccine)	Neutralizing antibody titers were similar for both vaccine arms (BA.1 vs. BA.4/BA.5). Titers against Omicron subvariants were lower compared to D614G. Higher titers against BA.4/BA.5 with the Wildtype/Omicron BA.4/BA.5 vaccine. All participants seropositive after boost.	Completed	No AESI, SAEs, or AEs leading to withdrawal. Most AEs were mild to moderate. Severe AEs were rare (1% local, 3% systemic). Lower titers observed in participants without prior infection.	NCT05289037

Effective processing of tumor antigens encoded by antigen-encoding mRNAs has shown promising results in vivo [9,37]. For this sake, encapsulated within biodegradable poly(β-amino ester) polymer NP formulation, mRNAs enter the cytoplasm of target cells via endosomal escape, where they are translated into antigen proteins, constituting the fundamental process for mRNA vaccine-based cancer treatment [19]. These antigens are then broken down into antigenic peptides in proteasomes. The Golgi apparatus and endoplasmic reticulum subsequently process these peptide epitopes into peptides, which follows their presentation to CD8^+^ T lymphocytes through the class I major histocompatibility complex (MHC-I) pathway [38]. On the other hand, mutations in cancer cells give rise to some antigenically novel antigens that are detected by CD4^+^ T lymphocytes as “nonself” via the MHC-II pathway. While several checkpoints present themselves as potential barriers to overcome during this process, for example, for an effective antigen processing, TAAs and tumor-specific neoepitopes need further optimization. A phase I trial (NCT03313778) is assessing a neoantigen-based LNP-encapsulated vaccine, mRNA-4157, which is estimated to be completed in the middle of 2025. However, an update of this study has shown low-grade reversible side effects of the mRNA-4157 vaccine, either alone or with pembrolizumab in 79 patients [39]. Notably, combination therapy demonstrated a higher response rate in HPV-negative head and neck squamous cell carcinoma patients compared to pembrolizumab alone, with longer progression-free survival. Another trial (NCT03897881), with a setting of 157 patients treated with mRNA-4157 plus pembrolizumab (*n* = 107) or pembrolizumab alone (*n* = 50), showed admirable results. The combinatorial therapy showed longer RFS, with fewer recurrence or death events. The grade ≥3 adverse events occurred in 25% of combination patients and 18% of monotherapy patients, with no severe mRNA-4157-related events [40].

Interestingly, specificity to tumor cells and the ability to elicit immune responses position the neoantigens as promising candidates for personalized treatment strategies. Personalized vaccines based on neoantigens have shown promising results in clinical trials, which demonstrate their ability to induce strong immune reactions [41]. However, ongoing research in neoantigen target discovery aims to enhance the identification of immunogenic neoepitopes recognized by CD8^+^ T cells [42]. This effort involves the development of advanced algorithms specifically tailored for predicting CD4^+^ T-cell neoepitopes, which present a more challenging task due to their diverse recognition mechanisms and complex interactions within the immune system [43]. These emerging algorithms leverage a combination of bioinformatics tools, machine learning techniques, and experimental validation to accurately predict CD4^+^ T-cell epitopes, thereby facilitating a comprehensive understanding of the immune response against cancer and opening new avenues for the development of novel immunotherapeutic strategies.

Researchers are currently developing novel mRNA vaccine platforms aimed at targeting a diverse array of tumor antigens. Personalized mRNA vaccines, specifically tailored to individual patients, have demonstrated promising efficacy in clinical trials [44]. These vaccines have shown promising results when combined with existing therapies, including immune checkpoint inhibitors and traditional treatments. For example, a phase I clinical trial (NCT05937295) is investigating the immunogenicity of the FusionVAC-22 peptide vaccine in combination with atezolizumab in fibrolamellar HCC harboring the DNAJB1-PRKACA fusion transcript [45]. This study estimates to be completed in 2027.

Another area of interest is to enhance the responses of immune cells to neoantigens in mRNA vaccines, such as the use of modified liposomes [46], improving the responses of exhausted T cells [47]. The application of mRNA modifications, such as pseudouridine, has shown superior stability and efficient translation [48].

Further research in mRNA cancer vaccines could explore optimizing antigen processing pathways and enhancing immune responses to neoantigens through various mRNA modifications. This could include investigating the use of modified liposomes and exploring different mRNA modifications in order to improve stability and translation. Additionally, ongoing trials could continue to assess the efficacy of personalized mRNA vaccines in combination with existing therapies across various cancer types. Moreover, the development of algorithms for predicting CD4^+^ T-cell neoepitopes remains crucial for advancing our understanding of immune responses and informing the design of novel immunotherapeutic strategies.

Clinical trials (summarized in Table 2) for mRNA vaccines are now underway as a cancer therapeutic option because of the promising outcomes of preclinical research. Along with neoantigens, dendritic cells (DCs) have shown promise in facilitating immune responses. DC-based vaccines rely on autologous DCs that are loaded with tumor antigens to stimulate an antitumor immune response [49]. mRNA vaccines have revolutionized this approach by allowing direct in vivo expression of tumor-specific antigens, thus eliminating the need for ex vivo DC manipulation [50]. While conventional DC-based vaccines involve antigen presentation through externally processed peptides [51], mRNA vaccines can directly encode neoantigens. This synergy between mRNA and DC-based vaccines has led to the development of novel immunotherapies that improve tumor-specific T-cell responses.

Therefore, DC-based vaccination, a widely explored immunotherapy method in clinical trials, involves a critical process. Patient-derived DCs are collected and then exposed to artificially manufactured autologous antigens. This step is essential in the manufacturing process of mRNA DC vaccines. By presenting these personalized antigens to immunocytes, the vaccine aims to activate targeted immune response against the specific tumor cells, potentially leading to enhanced antitumor effects [52]. Following this, the patient undergoes another infusion of DCs to activate and initiate an immunological response. One of the initial clinical trials for patients with advanced melanoma is the TriMix-DC vaccine (NCT01066390). This vaccine encodes multiple TAAs such as MAGE-A3, MAGE-C2, tyrosinase, or gp100, along with CD40 ligand and CD70, specifically designed for targeted therapy. A novel small LNP-based nanovaccine shows potent antitumor effects but can induce T-cell exhaustion. Combining with anti-PD-1 therapy, the regimen suppresses tumor recurrence, which highlights the importance of treatment timing in nanovaccine and immune checkpoint blockade combinations [53]. Further investigations are warranted to assess its suitability in clinical settings. Effective chemoimmunotherapy regimens are lacking; in a phase II trial (NCT03214250), nivolumab (nivo; anti-PD-1)/chemo met 1-year OS endpoint (57.7%), while sotigalimab (sotiga; CD40 agonistic antibody)/chemo (48.1%) and sotigalimab/nivolumab/chemo (41.3%) did not. Further research could focus on identifying patient subgroups that benefit most from the combination of nivolumab/chemo and exploring potential biomarkers for treatment response.

Neoantigens, tumor-specific mutations absent in normal tissues, play a crucial role in mRNA cancer vaccines by triggering targeted immune responses. Until recently, therapeutic cancer vaccines had not shown clear clinical benefits. However, recent trials—NCT03897881 in melanoma and NCT04161755 in pancreatic cancer—demonstrate promising outcomes. In NCT03897881, a phase IIb trial, combining immune checkpoint blockade with a neoantigen mRNA vaccine improved RFS compared to immune checkpoint blockade alone, though statistical power and follow-up time were limited. Similarly, in NCT04161755, pancreatic cancer patients who developed T-cell responses to the vaccine showed extended RFS. Despite small sample sizes and differences in baseline tumor characteristics, these findings highlight the potential of neoantigen mRNA vaccines in cancer immunotherapy.

Additionally, phase I evaluation of the TriMix-DC vaccine showed promising results in terms of immunogenicity and safety. Notably, strong CD8^+^ T-cell responses were observed when TriMix-DC vaccination was combined with the checkpoint inhibitor ipilimumab (NCT01302496).

Recent phase I trials have also investigated mRNA vaccines based on NPs. For example, BNT111, developed by BioNTech, showed positive outcomes in a phase I study. This vaccine encodes 4 TAAs antigens and targets metastatic melanoma. BNT111 has demonstrated an enhanced overall response rate when used in combination with cemiplimab, a PD-1 checkpoint inhibitor (NCT04526899). This combination therapy not only improves therapeutic efficacy but also maintains a favorable safety profile. Notably, BNT111 exhibits a well-tolerated safety profile both as a monotherapy and when administered alongside cemiplimab. Additionally, mRNA-based neoantigen vaccines are being fostered to further enhance immunogenicity. BNT122, currently in a phase II trial, delivers up to 20 neoantigens via intravenous (IV) administration and its evaluation is continued for efficacy and safety with pembrolizumab, a PD-L1 antibody. Additionally, phase II trial on mRNA-4157, which encodes up to 34 neoantigens, is currently under investigation against several solid tumors, including lung, melanoma, and colon cancer, both as a monotherapy and in combination with pembrolizumab. Several mRNA-based cancer vaccines have been studied in conjunction with immunotherapy and chemotherapy. For instance, a phase II trial combining BNT111 with cemiplimab (PD-1) is investigating its effectiveness in treating unresectable stage III or IV melanoma. Further, in a phase II trial (SOV01, NCT02107937), DCVAC, an autologous dendritic cell-based vaccine, improved progression-free survival in epithelial ovarian carcinoma patients. Analysis of 82 patients revealed that “cold” epithelial ovarian carcinomas with lower tumor mutational burden and low CD8^+^ T-cell infiltration responded better to DCVAC, suggesting potential personalized treatment strategies.

Currently, in an advanced HCC trial (NCT04251117), a personalized cancer vaccine was combined with pembrolizumab post-multityrosine kinase inhibitor therapy, demonstrating a safe profile with common injection-site reactions; the personalized therapeutic cancer vaccine encoded up to 40 neoantigens. Remarkably, 30.6% of patients responded, with complete responses linked to the number of encoded neoantigens, affirming the efficacy of PTCV in inducing neoantigen-specific T-cell responses [54]. Moreover, in a single-arm phase I/II study (NCT04212377) involving 7 patients with metastatic endometrial cancer, a DC vaccine was administered alongside chemotherapy (carboplatin/paclitaxel). The DC vaccine, containing Mucin-1 and Survivin antigens, was successfully produced in 5 patients, all completing the treatment regimen. Notably, antigen-specific responses were observed in 2 patients, while chemotherapy-related adverse events, predominantly neutropenia, were common.

In short, combining cancer nanovaccines with anti-PD-1 therapy reveals promising results in suppressing tumor recurrence, emphasizing the importance of treatment timing. Further exploration of patient subgroups benefiting from specific combinations like nivolumab/chemo is warranted, along with identifying biomarkers for treatment response. Additionally, ongoing trials evaluating mRNA vaccines and dendritic cell-based vaccines underscore the potential of personalized immunotherapy in various cancers, highlighting the need for further research in this domain.

## NP-Based mRNA Delivery

**Fig. 3.**
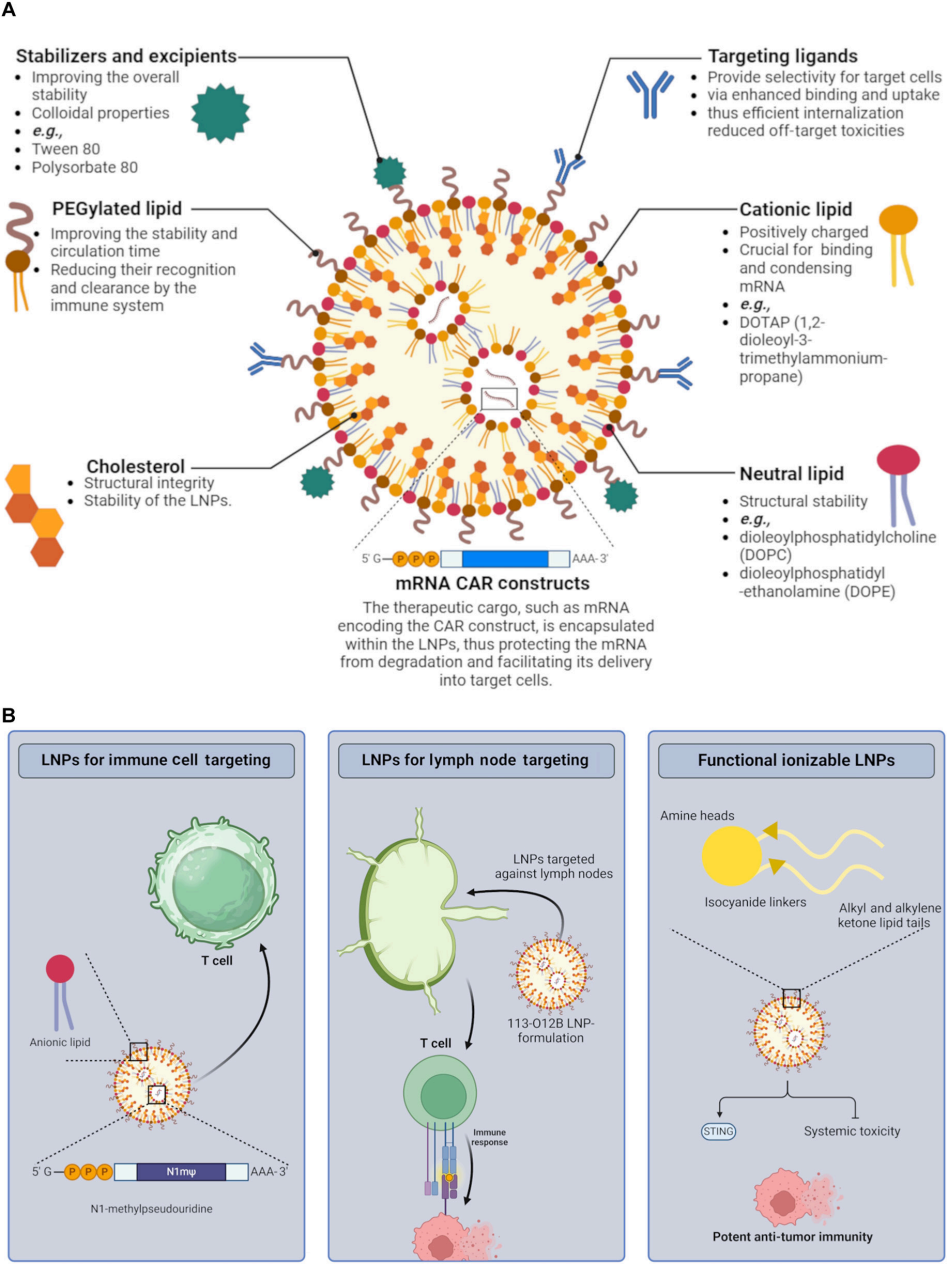
Design and mechanism of nanoparticle-based delivery of mRNA. (A) Cationic and neutral lipids stabilize and protect mRNA in LNPs, while PEGylation and targeting ligands enhance circulation, specificity, and therapeutic precision (reproduced from Khawar et al. [56]). (B) Immunocyte-targeted LNPs have been optimized to deliver mRNA specifically macrophages and T cells through chemical modifications, such as N1-methylpseudouridine (N1mψ), to enhance mRNA delivery and translation. Lymph-targeted LNPs emphasize the targeting of lymph nodes with specific LNP formulations, such as 113-O12B, which enhance mRNA delivery and show robust CD8^+^ T-cell responses against encoded antigens. Lastly, functional ionizable lipids for LNPs using a 3-component reaction improve mRNA transfection and activate immune pathways such as stimulator of interferon genes (STING), thereby leading to potent antitumor immunity.

One of the most advanced approaches for mRNA delivery involves encapsulating mRNA within LNPs, which are typically made up of 4 key components: (a) an ionizable or cationic lipid or polymer containing tertiary or quaternary amines, which encapsulate the negatively charged mRNA; (b) a zwitterionic lipid, such as 1,2-dioleoyl-sn-glycero-3-phosphoethanolamine, which mimics cell membrane lipids; (c) cholesterol, which stabilizes the lipid bilayer structure; and (d) a polyethylene glycol (PEG)-lipid, which forms a hydrating outer layer, enhances colloidal stability, and minimizes protein adsorption. A detailed design of LNPs is depicted in Fig. 3A. While the mechanism of LNP-mediated mRNA delivery is not yet fully understood, substantial progress has been achieved [55]. In a recent review, we discussed the advantages of LNPs over traditional methods and highlighted key considerations for modulating T-cell immune responses [56].

The effective use of mRNA-based therapeutics for cancer therapy is contingent upon the efficient exposure of mRNA to antigen-presenting cells, as well as the generation of a vigorous cytotoxic T-cell response. In this case, Ye et al. [57] have optimized LNPs for mRNA delivery to immune cells. Chemical modifications, especially N1mψ, considerably improved mRNA delivery and translation in macrophages supported by further validation in human primary T cells with notable cytotoxicity. The analysis of LNP properties emphasized the importance of anionic LNPs for macrophage penetration and the role of atom substitution in enhancing mRNA delivery to T cells [57]. Another study highlights the efficacy of LNPs targeted to lymph nodes for delivering mRNA in cancer vaccine development. 113-O12B LNPs demonstrated enhanced delivery to lymph nodes compared to others, resulting in improved CD8^+^ T-cell response to encoded antigens like ovalbumin and TRP-2 peptide. This led to enhanced protective and therapeutic effects against melanoma models, with a 40% complete response rate in B16F10 tumors when combined with PD-1 therapy [58]. Furthermore, methyl groups on the amine head and the ester linker were essential for mRNA distribution in vivo. Adjuvants can increase the effectiveness of mRNA vaccines since innate immunity is necessary for an effective immune response activation. Therefore, lymph node-targeting LNPs hold promise as a universal platform for next-generation mRNA vaccines with facilitation in long-term immune memory induction.

Miao et al. [59] employed a 3-component reaction to synthesize a diverse library of functional ionizable lipids with amine heads, alkyl and alkylene ketone lipid tails, and isocyanide linkers. In vitro testing revealed improved transfection compared to naked mRNA and in vivo analyses further confirmed efficient delivery to immune cells with potent lipids A2-Iso5-2DC18 and A12-Iso5-2DC18 showing enhanced antitumor immunity. Further investigations into the mechanism showed that these cyclic LNPs activate the stimulator of interferon genes (STING) pathway, inducing potent immune responses. Lead cyclic lipid A18 LNPs exhibit strong antitumor effects across cancer models, enhancing survival without systemic toxicity [59].

In recent years, organic and inorganic nanocarriers have been versatile tools used in mRNA delivery. These particles improve drug stability, target specific cells, and control release for better treatment outcomes in brain metastasis with fewer side effects [60]. Their customizable properties and surface modifications allow precise drug delivery, promoting precision medicine [61]. Additionally, nanocarriers can respond to environmental cues, enabling controlled drug release. They can target tumors passively via the enhanced permeability and retention effect or actively using ligands like antibodies to reach specific cells or tissues [62].

Recently, Younis et al. [63] demonstrated high mRNA encapsulation efficiency and p*K*_a_-dependent variations in particle size for pH-sensitive LNPs to activated hepatic stellate cells (HSCs). Lipids with higher p*K*_a_ values showed enhanced mRNA delivery to LX-2 cells. In a liver fibrosis model, nanocarriers, especially CL15A6, exhibited superior delivery to activated HSCs over hepatocytes, correlating with p*K*_a_. Manipulating particle size and N/P ratio influenced delivery efficiency. However, mechanistic elucidation highlighted factors like cellular uptake, hydrophobic scaffold of nanocarriers, and PDGFRβ receptor blockade affecting tropism of nanocarriers [63]. Later, Xiao et al. [64] in February 2024 optimized PEG linker length for PEGylated liposomes modified with mannose to efficiently delivery mRNA. The mannose receptor (CD206) on DCs was targeted for DC-targeted mRNA vaccines, utilizing mannose cholesterol with PEG linkers. mRNA-complexed mannose-modified liposome (MP*_n_*-LPX; where *n* = PEG linker lengths, i.e., 100/400/1,000/2,000) complexes showed efficient mRNA delivery to DCs, especially MP400-LPX, with enhanced transfection and antigen presentation abilities. MP400-LPX further demonstrated superior tumor inhibition and increased survival in murine models, highlighting its potential for effective tumor immunotherapy. Furthermore, MP400-LPX exhibited good safety profiles, suggesting its viability for systemic delivery and therapeutic applications.

Li et al. engineered bacterial outer membrane vesicles (OMVs) to incorporate RNA binding protein L7Ae and lysosomal escape protein listeriolysin O (OMV-LL), which aims to boost the effectiveness of mRNA vaccines for cancer treatment. OMV-LL enhances immune response by delivering mRNA to immune cells. In mice, OMV-LLmRNA OVA vaccine led to improved T lymphocyte activation with prolonged survival of tumor models, which reduces tumor growth and spread [65]. Huang et al. developed a series of alternating copolymers “PHTA” featured with *ortho*-hydroxy tertiary amine (HTA) repeating units using a one-pot amino epoxy ring-opening polymerization technique to deliver tumor antigen mRNA, avoiding interference with mRNA translation caused by certain cationic substances like cationic liposomes. These PNPs, particularly PHTA-C18, showed strong lymph node accumulation, negligible inflammatory effects, and considerable suppression of tumor progression (87% suppression effectivity) in melanoma, which indicates their potential for not only DC maturation stimulation but also T lymphocyte-mediated immune response against tumors [66].

In conclusion, optimized LNPs offer potential for immune cell delivery (Fig. 3B) [57,58], cyclic lipid LNPs activate STING pathway for antitumor effects [59], nanocarriers allow precise drug delivery [60,61], and recent studies stress NP design importance [63,64]. OMVs and PHTA-based PNPs show promise in boosting mRNA vaccine efficacy against tumors [65,66]. Future research should focus on combining various NP designs with adjuvants to enhance immune response activation. Investigating the mechanistic aspects of NP tropism, such as cellular uptake pathways and receptor interactions, could provide insights into improving targeted delivery. Moreover, assessing the long-term safety and efficacy of these advanced NP-based mRNA vaccines in larger animals and human trials would be crucial for their translation into clinical applications.

## mRNA-Based Antibodies

**Fig. 4.**
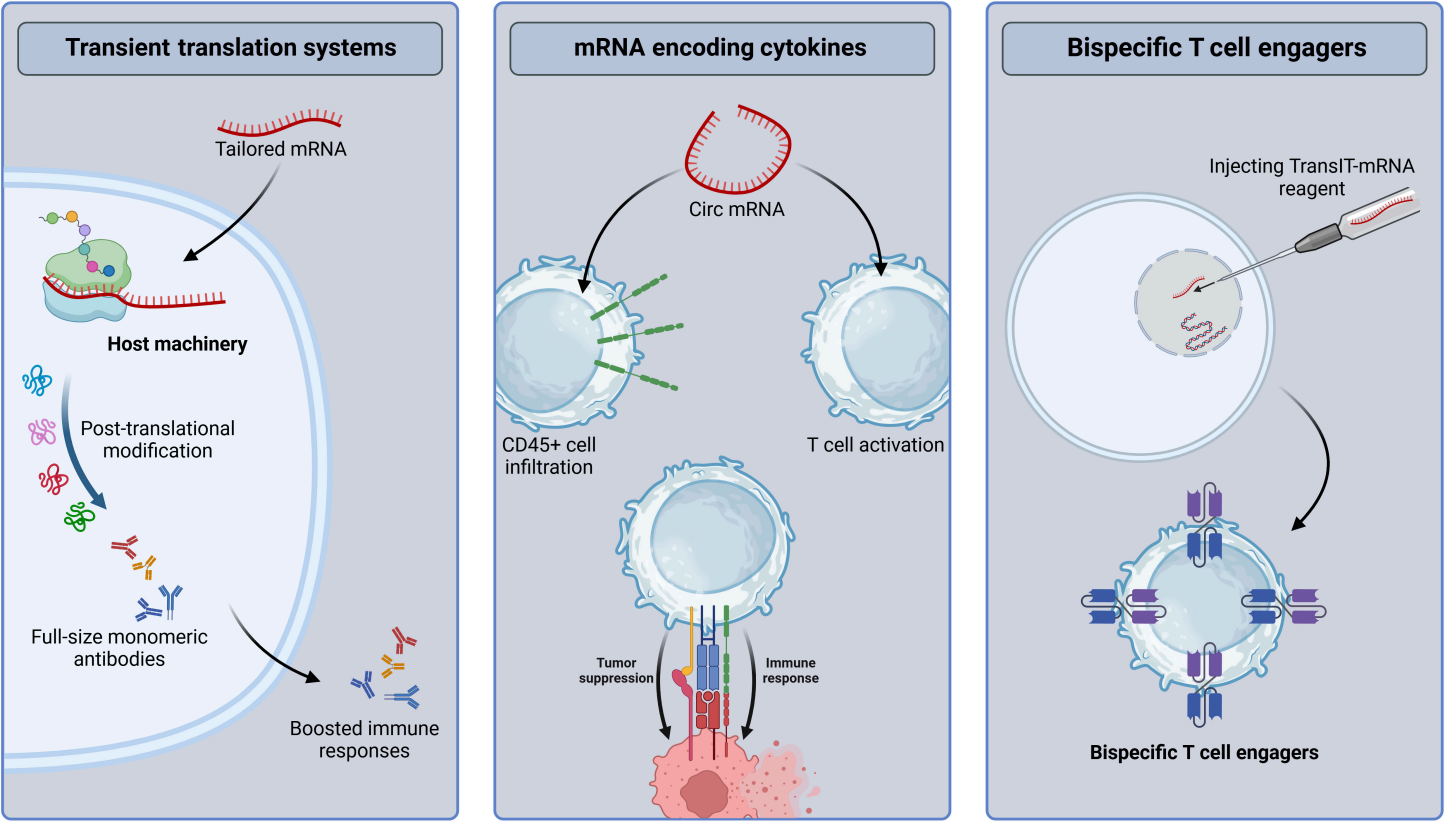
Mechanisms of mRNA-based antibody delivery. First, tailored mRNA is delivered to target cells, which utilize their own cellular machinery to translate the mRNA into the desired antibody proteins, ensuring proper assembly and posttranslational modification of full-size monomeric antibodies. Second, circular mRNA encoding cytokines is used to enhance T-cell activation within the TME, which promotes immune cell infiltration and activation and enhanced tumor suppression. Lastly, TransIT-mRNA transfection is employed to transport mRNAs that produce bispecific T cell-engaging antibodies in vivo, which activate polyclonal T cells to target and kill cancer cells through specific antigen–T cell interactions.

One of the most popular classes of biologics against cancer in the last few decades has been antibody-based therapies [67]. Numerous antibodies have been extensively studied against cancer, such as full-size monomeric antibodies, recombinant antibodies, and antibody fragments against cancer cells, immunocytes, and stromal cells in TME [68,69]. However, difficulties with using monoclonal antibodies (mAbs) and fragments have emerged, including the requirement to guarantee high concentrations and long-term persistence at tumor site. Using mRNA-encoding antibodies is a unique and exciting therapeutic option against cancer (Fig. 4). The correct assemblage and posttranslational modification of target cells are essential for full-size monomeric antibodies, which is ensured by delivering tailored mRNA to those cells and utilizing the translation system of host to make desired proteins [70]. This approach is utilized by Li et al. [34] whereby they present an innovative method for designing mRNA vaccines, integrating several adjuvants to boost immune responses. LNPs were utilized to deliver mRNA-encoded antigens efficiently, leading to an increase in SARS-CoV-2 antibody levels in mice. Incorporating a natural adjuvant from the C3 complement protein enhanced the effectiveness of vaccine, safety, and ease of administration, indicating the promising application of multiply adjuvanted mRNA nanotherapy in vaccination approaches. Another study has successfully delivered circular mRNA to encode cytokines [71] where they showed that the cytokine-encoding circular mRNA enhances T-cell activation in the TME for tumor suppression. Analysis of posttreatment tumor-infiltrating T cells revealed increased CD45^+^ cell infiltration with circular mRNA treatment, particularly in conjunction with anti-PD-1 antibody, which indicates enhanced immune cell influx. Additionally, combination therapy has elevated tumor-infiltrating T lymphocytes, thereby enhancing T lymphocyte infiltration and activation.

Overexpression of human epidermal growth factor receptor 2 (HER2) causes cellular overstimulation, which eventually results in abnormally fast growth and malignant proliferation. It is pertinent to note that HER2 expression varies among various cancers, as evident by Shayed et al., when they conducted a multi-institutional testing of HER2 across diverse cancers. The results indicated diverse rates of HER2 amplification, mRNA overexpression, and positive immunohistochemistry. Notably, some patients with HER2 mRNA positivity, despite insufficient tissue for immunohistochemistry and copy number variation assessment, showed responses to anti-HER2 therapy [72].

A common mAb used in the clinic that targets HER2, trastuzumab, has been demonstrated to successfully slow the proliferation of tumors. Similarly, Rybakova et al. [70], successfully generated trastuzumab in vivo using anti-HER2 mRNA-loaded LNPs to target liver in mouse models. An IV administration of 2 mg/kg anti-HER2 mRNA-loaded LNPs resulted in almost 40 mg/ml of trastuzumab in 24 h, maintaining levels around 45 ± 8.6 mg/ml for 2 weeks [70]. Recently, HER3-directed antibody patritumab deruxtecan treatment in 77 patients led to a considerable increase in CelTIL score, with 45% overall response rate observed; genomic changes included a shift toward less proliferative tumor phenotypes and upregulation of immunity-associated genes, with treatment-emergent adverse events reported in majority of patients [73].

Anti-HER2 mRNA injections prolong the lifespan of mice by delaying HER2-positive tumor formation, while bispecific T-cell engager antibodies activate polyclonal T cells to target and kill cancer cells through specific antigen–T cell interactions [74]. For this case, Stadler et al. [75] have used TransIT-mRNA transfection to transport mRNAs in order to produce bispecific T cell-engaging antibodies in vivo to assess the potential of LNP carrying bispecific T-cell engagers–mRNA, which markedly reduced the growth of tumors. Based on encouraging preclinical results, 2 mRNA-based antibody treatments are currently in clinical evaluation for treating solid tumors. In the phase I/II trial (NCT04683939), BNT-141, an mRNA-based therapy encoding an IgG antibody, is being rigorously tested to evaluate its safety profiles, pharmacokinetic characteristics, and preliminary efficacy. This trial is particularly focused on patients suffering from metastatic or unresectable tumors that express CLDN18. Concurrently, another phase I trial (NCT05262530) is investigating BNT-142, an innovative mRNA therapy designed to produce BiTEs (bispecific T-cell engagers) targeting both CLDN6 and CD3. The primary goals of this trial are to determine the safety and preliminary efficacy of BNT-142 in patients with solid tumors expressing CLDN6. Both trials represent important steps forward in the development of mRNA-based treatments for solid tumors.

The current trend is shifting toward wide applications of anti-HER2 therapy where, in preclinical settings, a sufficient progress has been made by Li et al. [76] as they identified membrane-localized HER2 expression in melanoma cells, with RC48 effectively targeting HER2-positive melanoma while inducing tumor regression in combination with dabrafenib.

In brief, the exploration of mRNA-based antibody therapies against cancer, exemplified by fresh advancements in HER2-targeted therapies and bispecific T-cell engagers, underscores a promising avenue for improving patient outcomes. Future research should focus on expanding clinical trials of mRNA therapies targeting specific antigens and exploring synergistic treatment combinations to enhance therapeutic efficacy and minimize adverse effects.

## mRNA-Based Costimulatory Agents

T-cell activation and function is regulated by costimulatory domain molecules on cell surfaces, which influence their specific responses to antigens. In cancer treatment, reduced levels of costimulatory molecules in tumor-infiltrating immune cells hinder effective immune responses. Therefore, developing strategies to enhance costimulation in T cells is critically important, aiming to improve antitumor immune responses and overcome immune evasion by tumor cells. Interestingly, combining these approaches with immune checkpoint inhibitors shows promise for enhancing cancer immunotherapy and requires further exploration to maximize effectiveness [77]. In response to the rising interest in mRNA-based costimulatory agents for cancer therapy, researchers have developed nanomaterials targeting immune cells for modulation. Li et al. [78] developed PL1, an NP delivering OX40 mRNA to boost its expression in T cells. PL1-OX40 mRNA delivery in combination with anti-OX40 antibody treatment showed slowed tumor growth effectively. PL1-CD137 mRNA reduced tumor growth in melanoma and lymphoma models. PL1-OX40 mRNA treatment decreased tumor growth and prolonged survival across various models, with some mice showing complete regression. PL1-OX40 treatment increased OX40 expression on tumor-infiltrating lymphocytes and elevated cytokine levels associated with antitumor immunity. Combining PL1-OX40 with immune checkpoint inhibitors enhanced tumor regression synergistically [78].

**Fig. 5.**
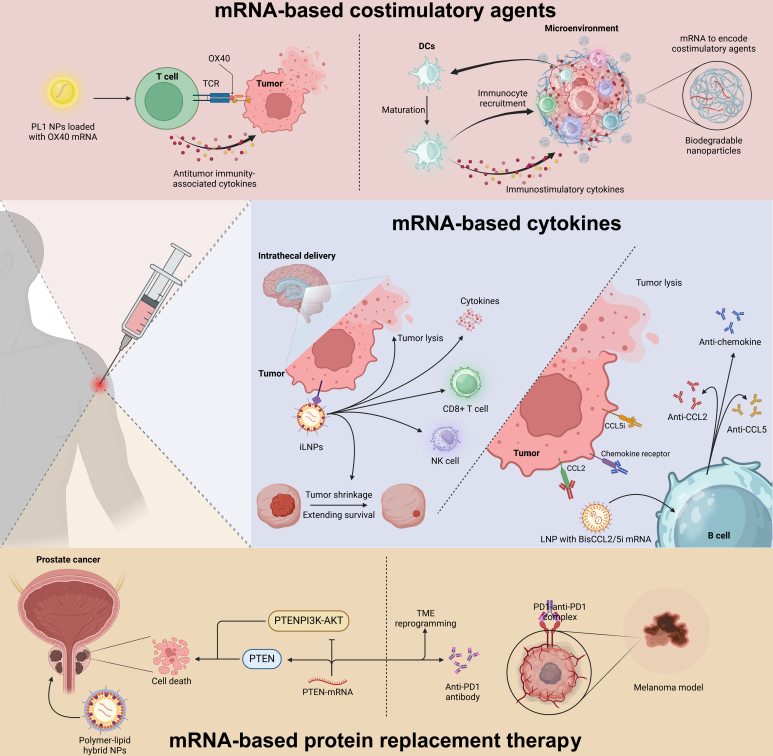
Mechanisms of mRNA-based therapeutic agents for tumor immunomodulation. PL1 nanoparticles deliver OX40 mRNA to T cells via enhancing OX40 expression and boosting cytokine levels and synergizing with immune checkpoint inhibitors for tumor regression. Biodegradable nanoparticles deliver mRNA encoding immune-stimulatory molecules directly into tumors, which induce dendritic cell maturation, and reprogramming the TME for sustained tumor regression and resistance to rechallenge. For mRNA-based cytokines, ionizable LNPs encapsulate cytokine mRNAs, which target tumor cells intrathecally to reduce tumor progression and increase immune cell infiltration. Combination with anti-PD-1 therapy enhances antitumor responses. LNP-formulated mRNA expressing BisCCL2/5i targets TAMs, reducing immunosuppression and polarizing TAMs toward an anticancer M1 phenotype to improve survival with PD-L1 therapy in cancer models. Lastly, for mRNA-based protein replacement therapy, PTEN-mRNA nanotherapy uses polymer–lipid hybrid nanoparticles to restore PTEN expression and inhibit PI3K-AKT pathway while inducing tumor cell death. Combining PTEN-mRNA with PD1 antibody therapy enhances anticancer effects and reprograms the TME to become effective in models of PTEN-mutated melanoma and PTEN-null prostate cancer.

Recently, researchers have used biodegradable NPs to deliver mRNA encoding immune-stimulatory molecules directly into tumors to reprogram the TME for enhanced immunostimulation (Fig. 5). This approach induced sustained tumor regression, synergized with immune checkpoint blockade, and conferred resistance to tumor rechallenge at distant sites by boosting immunostimulatory cytokine production and immune cell recruitment [79]. Delivery of costimulatory domains via mRNA induces dendritic cell maturation, leading to distinct transcriptional programs. TriMix mRNA triggers an antibacterial response, while TetraMix mRNA redirects DCs to an antiviral program. TetraMixDCs show potential in generating tumor antigen-specific T cells, shifting from naive to memory T cell subsets with cytotoxic capacity, suggesting a promising strategy for antitumor immune activation in cancer patients. Recent data show that the delivery of costimulatory domains via mRNA induces DC maturation, which leads to distinct transcriptional programs. TriMix mRNA triggers an antibacterial response, while TetraMix mRNA redirects DCs to an antiviral program. TetraMixDCs show potential in generating tumor antigen-specific T cells, shifting from naive to memory T-cell subsets with cytotoxic capacity [80].

Incorporating mRNA-based costimulatory agents in cancer therapy exhibits promising outcomes, with nanomaterial delivery systems like PL1 effectively boosting T-cell responses and enhancing tumor regression, particularly when combined with immune checkpoint inhibitors. Furthermore, recent advancements utilizing biodegradable NPs to deliver mRNA encoding immune-stimulatory molecules directly into tumors show sustained tumor regression and enhanced immunostimulation. These insights hold promise for reshaping the TME and generating antitumor immune responses.

## mRNA-Based Cytokines

Cytokines are crucial players in immune responses and act as messenger among various immunocytes. Lymphocytes, macrophages, and DCs, along with stromal cells, frequently produce these molecules within the TME. This production establishes a unique cytokine milieu capable of either stimulating or inhibiting tumor growth as per the specific cytokine present, or concentration of specific cytokine [81,82].

For example, certain cytokines like interleukin-2 (IL-2), IFN-γ, and IL-12 have been found to inhibit tumor growth [83,84]. Conversely, interleukins, tumor necrosis factor-α (TNF-α), TGF-β, interferons, and vascular endothelial growth factor (VEGF) are among the specific cytokines identified to promote tumor growth [85]. Furthermore, cancer-associated fibroblasts secrete cytokines like activin A, IL-5, and angiogenin, which have been demonstrated to enhance tumor growth in breast cancer [86].

Owing to its ability to trigger immune responses within tumors, cytokine-based therapy has appeared as a potential therapeutic option and remains a focal point of current clinical research. Preclinical studies have shown the efficacy of interleukins, IFN-α, and GMCS factor in combating various malignancies in mouse models [87–89]. The US FDA has approved the application of IL-2 and IFN-α against melanoma and renal cell carcinoma [90,91]. These cytokine medications are being used less commonly, nevertheless. This is largely because when used systemically, cytokines are frequently linked to serious dose-limited toxicities and have not demonstrated any clinical benefit in monotherapy [92]. A growing body of research indicates that administering cytokines that encode mRNA is a safe method of treating cancer. In several murine tumor models, a recent preclinical investigation showed that intratumoral saline-formulated mRNAs are delivered to encode IL-12, IFN-α, granulocyte-macrophage colony-stimulating factor (GM-CSF), and IL-15 to immunize against tumor. Moreover, treatment with anti-PD-1 and mRNA nanotherapy together improved antitumor responses and increased tumor shrinkage, extending the lifetime of the mice [11]. Liu et al. [92] created ionizable LNPs encapsulating cytokine encoding mRNAs, including GM-CSF, IL-12, and IL-27, in an effort to increase the therapeutic efficacy even further. Intrathecal delivery of IL-12 and IL-27 genes via these LNPs was shown to radically reduce B16F10 melanoma progression with statistically nonsignificant off-tumor toxicity [92]. In the meantime, following mRNA nanotherapy, there was a strong influx of effector immunocytes, including NK and CD8^+^ T lymphocytes, in TME, which suggests that these ionizable LNPs successfully transported cytokines encoding mRNAs to the tumor cells to induce immunity against tumors (Fig. 5). Apart from cytokines, chemokines have the ability to stimulate APC maturation and activation, trigger T cell-mediated immunity, and govern the malfunctioning tumor TME. For these reasons, their use in cancer immunotherapy has garnered an ample interest. Immunosuppression is induced in part by tumor-associated macrophages (TAMs), which are substantially abundant in the TME. Two chemokines that are specifically chosen to draw TAM infiltration as well as cause polarization toward the tumor-inducing M2 phenotype are CCL2 and CCL5 [93]. Wang et al. [94] created LNP-formulated mRNA to express antibodies for these aforementioned phenotypes, known as BisCCL2/5i, to enhance M1-mediated anticancer effects. The immunosuppression in TME was lessened by this BisCCL2/5i mRNA nanotherapy, which also strongly increased TAM polarization toward an anticancer M1 phenotype. Further evidence came from in vivo studies showing that BisCCL2/5i mRNA and PD-L1 together increased mouse survival among a variety of hepatic and pancreas cancer models. To date, over 5 mRNA-based cytokine treatments pave their way in clinics. For instance, individuals with metastatic or incurable solid tumors have been treated with BNT-151, both alone and in conjunction with other anticancer medications (NCT04455620). LNPs encapsulating IL-7 and IL-2 mRNAs are used in BNT-152 and BNT-153 clinical trials to examine the efficacy and safety in various solid malignancies in subjects (NCT04710043 and NCT04710043). In advanced solid tumor patients, BNT-131 (SAR441000) mRNAs that encode IL-12, IFN-α, GM-CSF, and IL-15 are now being studied as a monotherapy also in conjunction with cemiplimab (NCT03871348). The China Center for Drug Evaluation has approved SW0715 of StemiRNA, an LPP-formulated mRNA encoding the cytokine IL-12, to start a clinical trial treating breast cancer and melanoma alongside PD-1 mAb.

In short, cytokines such as IL-2, IFN-γ, and IL-12 demonstrate tumor-inhibitory effects; however, others like TNF-α, TGF-β, and VEGF often contribute to tumor progression. This dichotomy emphasizes the need for precise, context-dependent delivery systems to harness their therapeutic potential effectively. Currently, researchers have achieved localized, controlled expression of these potent immune modulators by encoding cytokines such as IL-12, IFN-α, GM-CSF, and IL-15 within mRNA formulations while minimizing systemic toxicity and enhancing antitumor immunity. Beyond cytokines, the role of chemokines in reshaping the TME has garnered considerable attention. For instance, mRNA-based chemokine therapies, such as BisCCL2/5i, have demonstrated the ability to reverse immunosuppression and enhance anticancer immunity. These advancements, coupled with the ongoing clinical evaluation of mRNA-based cytokine therapies like BNT-151, BNT-152, and SW0715, underscore the promise for cancer treatment; however, challenges remain.

The optimization of mRNA delivery systems, the mitigation of off-target effects, and the exploration of synergistic combination therapies are critical areas for future research. As the field progresses, the integration of mRNA-based cytokine therapies with other immunomodulatory strategies, such as immune checkpoint blockade and adoptive cell therapy, holds immense promise for achieving durable antitumor responses. By addressing these challenges and leveraging the unique advantages of mRNA technology, cytokine-based cancer immunotherapy may soon transition from a promising experimental approach to a cornerstone of modern oncology.

## mRNA-Based Protein Replacement Therapy

Currently, a revolutionary approach to treating genetic diseases is emerging, one that harnesses cellular machinery to synthesize therapeutic proteins. Delivering mRNA conveys precise instructions to cells, thus transforming them into miniature factories that produce those proteins missing or dysfunctioning due to genetic mutations.

Interestingly, mRNA-based protein replacement therapy is emerging as a groundbreaking approach that leverages mRNA to instruct cells to produce specific therapeutic proteins that may be missing or dysfunctional. Unlike traditional gene therapy, which involves modifying the DNA, mRNA-based therapy provides a transient and controlled production of proteins, reducing the risks associated with permanent genetic alterations. This approach holds promise for treating a wide range of diseases, including enzyme deficiencies, hemophilia, and certain types of cancer, marking an important advancement in personalized medicine [95,96]. Nonetheless, exogenous administration of therapeutic proteins faces various challenges, for example, reduced delivery, lower localization to cellular or subcellular compartments, challenges in their manufacturing therapeutic proteins, and ultimately higher costs.

Thus, therapeutic proteins undergoing clinical trials encounter several challenges, which include insufficient concentrations at target sites and reduced cellular uptake owing to their higher molecular weight, which often exceeds 100 kDa [96,97], the intricate in vitro environment [98–100], and immune clearance, among other issues [97]. Owing to mRNA engineering advancements, mRNA-based treatments have appeared to be an attractive technique for the manufacturing of therapeutic proteins in vivo against solid tumors, genetic disorders, and cardiovascular illnesses [101–103]. Earlier, Jirikowski et al. [104] effectively treated uremia in animal models via IV administration of brain-derived pure mRNAs, which was regarded as a pioneering work in mRNA-based therapeutics. Given these challenges, administration of mRNA via various delivery platforms to produce proteins of therapeutic or cytotoxic properties, for instance, antibodies, chemokines, tumor inhibitors, or other some costimulatory agents, is necessary. This innovative approach is supported by promising preclinical and clinical research that indicates enhanced safety with antitumor efficacy in comparison with conventional protein-based treatments [97,105]. By harnessing the cellular machinery to produce these critical proteins, this strategy holds major potential for improving cancer therapy outcomes.

Tumor suppressors delivered via mRNA nanotherapy can help restore the function of lost or mutated tumor suppressor genes, which are frequently observed in numerous human malignancies [10]. These tumor suppressors, which include RB1 [106–108], phosphatase and tensin homolog deleted on chromosome 10 (PTEN) [109], and p53 [110], were frequently involved in the onset, progression, and resistance to treatment of cancer. Restoring the tumor suppressor expressions or forcing tumor cells to manufacture deadly cytoplasmic proteins that cause cell lysis are 2 possible uses for mRNA [111]. Many mRNA-encoding tumor-suppressor NPs have been produced for this strategy, and their effects on cancer therapy have been encouraging. For example, a polymer–lipid hybrid NP was described by Islam et al. [111] for delivering PTEN-mRNA to PTEN-null prostate cancer in vivo (Fig. 5). Poly lactic-co-glycolic acid (PLGA), lipid-PEG, and cationic lipidoid chemicals G0-C14 made up the hybrid NPs. PLGA stabilizes NP core, while G0-C14 was used for the encapsulation of mRNA. Results demonstrated that PTEN-mRNA NPs induced restoration leading to in vitro and in vivo cell lysis. According to mechanistic research, the PI3K-AKT pathway was inhibited when PTEN protein was restored. This is supported by the fact that PI3K-AKT is crucial for controlling the cell cycle, apoptosis, and cell proliferation.

Lin et al. [112] created a redox-responsive LNPs composed of a co-polymer with G0-C14 and a GSH-responsive unit to enhance the translational efficiency of PTEN-mRNA. Results showed efficient delivery of PTEN-mRNA via modified LNPs to the tumor site where it aids in the lysosomal release of PTEN-mRNA. The novel PTEN encoding mRNA nanotherapy effectively inhibited tumor development and CD8^+^ T lymphocyte infiltration within TME in vitro. Recent studies have highlighted the potential of combining genetic and immunotherapy approaches in cancer treatment. For instance, PTEN-mRNA nanotherapy, when used alongside a PD-1 antibody, has shown a remarkably potent anticancer effect in both a subcutaneous model of PTEN-mutated melanoma and an orthotopic model of PTEN-null prostate cancer. Likewise, to transport p53 mRNA to tumor hepatocytes in vivo, Xiao et al. [113] created targeted LNPs made of G0-C14, PLGA, and lipid-PEG. In studies on HCC models, the combination of p53-mRNA nanotherapy and anti-PD-1 therapy showed better results than either treatment alone. This combined approach not only increased p53 expression but also reprogrammed the TME, which enhanced the overall antitumor effects. In the meantime, a number of redox-responsive NPs have been engineered for an efficient mRNA delivery that encodes tumor suppressor, which has been shown to be effective in discontinuing the tumor progression via inducing not only the arrest of cell cycle but also autolysis [10]. Tumor protein editing can also be accomplished by mRNA-based nanotherapy. For instance, DUF5 mRNA was delivered into tumor cells by Cai et al. [114] using a biodegradable LNPs in order to delete mutant RAS proteins. Because LNPs prefer to transport mRNA to cancer cells over normal cells, mRNA nanotherapy has demonstrated the ability to cure cancer by effective inhibition of mutant RAS signaling. Nevertheless, following systemic administration, the majority of NP-based delivery cargos show strong propensity to deposition in hepatocytes and might be potentially hepatotoxic [115–117]. In particular, it is critical that the proteins toxic to cells exclusively express in targeted cancer cells. In order to tackle this problem, Jain et al. [118] put forth a fascinating plan that involves adding target sites of miRNA into mRNAs to down-regulate it in normal hepatocytes but up-regulate it in HCC cells. The systemic delivery of mRNA-based nanotherapy may benefit greatly from this miRNA-mediated eradication of hepatotoxicity.

In short, mRNA-based protein replacement therapy has emerged as a promising approach, addressing limitations of traditional protein-based therapies in cancer, genetic disorders, and other diseases. Future directions should explore optimizing mRNA delivery systems, enhancing tumor targeting specificity, and combining mRNA therapies with other treatment modalities for improved clinical outcomes.

## mRNA-Based Adoptive Cell Therapy

The application of engineered CAR therapy is a novel and potentially effective treatment for several hematological cancers [119]. Moreover, there has been a lot of interest in nanoimmunoengineering of CAR T cells via mRNA delivery [19]. Earlier, we discussed barriers for CAR T cells and strategies to mitigate the CAR-related toxicities [120]. However, despite an extensive research in the area, the focus of scientific community is diverting toward alternative methods owing to the adverse effects of CAR T cells [121]. These alternative strategies include CAR NK and CAR macrophage engineering [122] to further overcome the CAR-associated challenges in T cells [121]. The therapy involves genetically altering either autologous or allogenic T cells to produce CARs [123,124]. Upon encountering cancer cells displaying the specific antigen, CAR T cells utilize their engineered receptors to bind directly to this antigen on the cancer cell surface. Following this binding event between the CAR and its specific antigen on the target cancer cell, a critical downstream signaling cascade is initiated [125,126]. This antigen recognition triggers the activation of the CAR T cell, priming it for a targeted attack against the cancer cell. The design of CARs allows for a high degree of customization, enabling them to recognize a broad array of tumor antigens. Researchers are actively exploring various CAR configurations in preclinical and clinical settings. These configurations focus on optimizing different functional domains within the CAR molecule, and this tailored design approach allows for the development of CAR T cells with enhanced specificity and potency against specific tumor types. The remarkable ability of CAR T cell therapy to harness the immune system to combat cancer has led to its successful application in treating certain types of tumors, particularly blood malignancies. This targeted immunotherapy approach holds immense promise for revolutionizing cancer therapy [127]. Presently, virus-mediated delivery of viral gene transduction is the most common technique for producing CAR-engineered T cells. On the other hand, effector T-cell genotoxicity and insertional mutagenesis are risks associated with viral vectors. Furthermore, the cell cycle phase of T lymphocytes has a significant impact on the efficacy of viral vector-based transduction [128,129]. As a result, new nanotechnology methods for modifying T cells are presently being investigated. The unique properties of mRNA render it a promising tool for T-cell engineering. Delivery methods for mRNA in cell engineering are continually evolving, with nanocarriers emerging as particularly advantageous for immune cell applications. These nanocarriers offer improved cellular uptake via specifically modifying the surface using proper ligands. Additionally, they hold the promise in combinatorial therapy as multiple therapeutic payloads can be loaded within a single carrier. This versatility positions mRNA nanotherapy as a highly suitable platform for generating CAR T cells, CAR natural killer (NK) cells, and CAR macrophages. Notably, a major challenge in genetically engineering innate immune cells, including T cells, macrophages, and NK cells, stems from their inherent resistance to traditional viral vectors. Tumor regression has been improved with enhanced nonviral NP formulations, which have shown greater effectiveness in mRNA delivery and CAR expression. Reinhard et al. [130] engineered T lymphocytes using an N-[1-(2,3-dioleyloxy)propyl]-N,N,N-trimethylammonium chloride-based LNP to deliver mRNA encoding an scFv that targets claudin 6 on tumor cells surface. In a CT26 colon cancer animal model, CAR T lymphocytes were demonstrated to induce regression of resistant tumor. On the other hand, Billingsley et al. [131] identified a new protocol and used C14-4 ionizable LNPs for mRNA delivery to human T lymphocytes, which is less damaging to T cells compared to the commonly used electroporation technique. The findings indicated the promising use of the very strategy since the LNP encapsulating mRNA can aid transfecting T lymphocytes effectively, thereby inducing CAR expression, which improved T lymphocyte-mediated targeting of tumors in vivo and in vitro. In addition, the authors created a methodical technique for LNP optimization that is utilized in CAR-T cell mRNA engineering [132]. They evaluated their LNPs composition with amine groups and optimized the efficiency of delivering mRNA to CAR T lymphocytes using an orthogonal design of experiments (DoE) approach. These LNP formulations were found to successfully transfect mRNAs to encode CARs into T lymphocytes ex vivo and in vivo. Given the high costs and rigorous procedures involved in ex vivo T-cell engineering, in vivo T-cell programming offers a practical alternative. Consequently, Parayath and researchers developed injectable LNPs of PEGylated lipids and ionizable lipids with amine groups attached. These formulations have shown promise for delivering IVT mRNA-encoded receptors to T lymphocytes in vivo [133]. Recently, we reviewed mRNA delivery via LNPs that can transiently express CARs on circulating T cells. Future hotspots such as efficient gene transfection to immunocytes, desired organ distribution, preventing accumulation in liver, and premature degradation are crucial in leading mRNA delivery via LNPs to the clinical settings [56]. In addition to T-cell manipulation, the engineering of other immunocytes, for example, macrophages and NK cells, have been investigated for cellular therapy. For instance, Ye et al. [57] demonstrated that LNP formulation is used to engineer macrophages by delivering CAR mRNA into M1 macrophages with an inflammatory phenotype. The LNP formulation has shown transfection efficiencies of 51.3% for Mφ cells and 22.5% for M1 macrophages. In vitro experiments revealed that CAR-Mφ and CAR-M1 cells achieved killing efficiencies of 32.54% and 22.50%, respectively, against human B lymphoma cells. Building on prior research, Zhang et al. presented a novel approach utilizing PBAE polymer NPs for targeted delivery. This system reprograms M2 macrophages toward the M1 phenotype in vivo through the delivery of IRF5/IKKβs mRNA [134]. Similarly, preclinical data indicated that IRF5/IKKβ-encoding NPs were administered intraperitoneally for ovarian carcinoma, intravenously for melanoma lung cancer, and intracerebrally for spongioblastoma. These approaches considerably slowed tumor growth while, in other cases, completely eradicating the tumors in the animals [134]. In clinical studies, mRNA nanotherapy has been administered to individuals with ovarian cancer as a direct monotherapy through an intraperitoneal catheter, inspired by promising preclinical results [56].

**Fig. 6.**
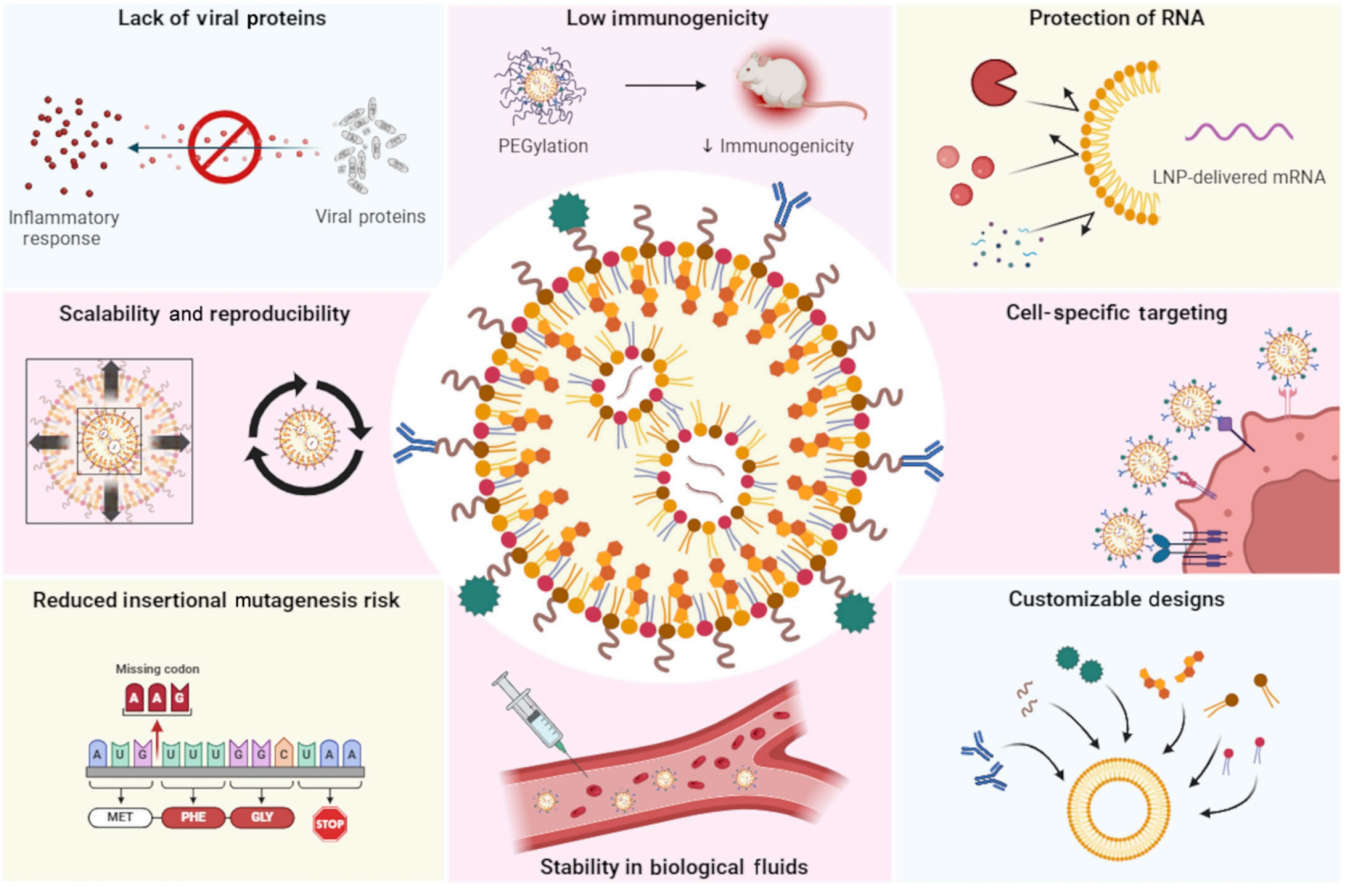
Safety and efficiency of LNPs in delivering CAR-mRNA constructs for mRNA-based adoptive cell therapy. LNPs are a safer and more efficient method for delivering CAR-mRNA constructs in mRNA-based adoptive cell therapy. Their lower immunogenicity, stability, and ability to protect mRNA while enabling cell-specific targeting make them a promising platform for clinical applications (reproduced from Khawar et al. [56]).

In conclusion, the field of CAR-based immunotherapy is rapidly evolving, with substantial strides made in overcoming the limitations of traditional CAR T-cell therapy. To mitigate viral transduction-associated risks, such as genotoxicity and insertional mutagenesis, LNPs have emerged as a promising platform (Fig. 6) for the transient and efficient delivery of CAR-encoding mRNA to T cells, macrophages, and NK cells. Furthermore, the ability to engineer innate immune cells, such as macrophages, to target tumors underscores the versatility of mRNA nanotherapy. Despite these breakthroughs, challenges remain, including optimizing gene transfection efficiency, achieving targeted organ distribution, and minimizing off-target effects. As research continues to refine these delivery systems, mRNA-based CAR therapies hold immense potential to revolutionize cancer treatment, offering a safer, more adaptable, and cost-effective approach to harnessing the immune system against malignancies. The integration of combinatorial strategies and the exploration of novel NP formulations will be pivotal in translating these innovations from preclinical success to clinical reality.

## mRNA-Based Gene Editing via the CRISPR-Cas9 System

Gene editing involves precisely altering the DNA within a cell by deleting, inserting, or replacing specific segments to rectify genetic mutations or induce new genetic traits. This technique allows for targeted modifications, enabling researchers to address genetic disorders or enhance cellular functions by making precise changes to the genome [135–137]. The discovery of the CRISPR-Cas9 system, which stands for clustered regularly interspaced short palindromic repeats and associated protein 9, marked a major breakthrough in genetic engineering. This innovative system, derived from a natural defense mechanism in bacteria, enables precise editing of DNA by creating targeted double-strand breaks (DSBs) at specific genomic locations. The subsequent repair processes can be harnessed to insert, delete, or modify genetic material, providing a powerful tool for a wide range of applications in research, medicine, and biotechnology [138,139]. This breakthrough has advanced the feasibility of various gene editing applications, such as genome editing, gene therapy, and cell therapy.

The CRISPR-Cas9 system is composed of a single guide RNA (sgRNA) and a Cas9. Subsequently, sgRNA directs Cas9 to the particular locations on DNA, where it induces DSBs. These breaks are then repaired by the nonhomologous end-joining (NHEJ) repair mechanisms, which are error-prone and can lead to gene insertions or deletions [138]. For effective genetic editing in vivo, the CRISPR-Cas9 system must be delivered to target cells. Protein, plasmid DNA, or mRNA can all be used to deliver Cas9 [117]. However, delivering plasmid DNA involves overcoming the barriers of membranes. The Cas9 protein of 160 kDa poses major challenges for in vivo distribution using nonviral delivery methods. In contrast, mRNA can be translated into Cas9 after merely crossing the cell membrane, which makes it possible to co-deliver Cas9 mRNA and sgRNA. According to Ibba et al. and Baptista et al. [117,140], this method leverages the efficiency of mRNA translation to facilitate gene editing processes more effectively. Moreover, an advantage of mRNA-based CRISPR-Cas9 is its ability to be delivered into tumor cells without using viral vectors. This approach can potentially make mRNA-based gene editing more effective and safe compared to using viral delivery [141]. mRNA-based gene editing offers the advantage of temporarily modifying protein expression within a cell, in contrast to viral gene editing resulting in permanent alterations to a genetic makeup. This temporary nature of mRNA-based editing allows researchers to evaluate the effects of genetic modifications without making irreversible changes to the DNA sequence. As a result, it provides a valuable tool for studying gene function and assessing the impact of potential therapies with reduced risk of long-term genetic alterations.

Tumorigenesis is typically caused by a specific genetic mutation, and conventional therapy employing targeted inhibitors may result in resistance to drugs and poor outcome in clinics. Gene editing with mRNA has the potential to fix mutation while providing persistent advantages. CRISPR-Cas9-mediated editing shows potential in its ability to permanently knock out genes encoding inhibitory receptors on T lymphocyte or tumor cells, for example, PD-1, PD-L1, and CTLA-4. Additionally, it can interrupt genes crucial for survival or those responsible for resistance to drugs. This capability highlights its potential for developing more effective cancer treatments that could potentially circumvent the drawbacks of repeatedly administering conventional cancer treatments and increase their efficacy [142]. Delivering CRISPR-Cas9 mRNA to targeted cells and overcoming various intracellular and extracellular obstacles could be achieved using NP-based platforms. These platforms offer high efficiency in cytosolic transport and can be engineered to target specific organs or cell types, enhancing the precision and effectiveness of gene editing. In light of this, Rosenblum et al. [142] created an effective nonviral LNP method for CRISPR-Cas9 mRNA delivery that targets tumors. Their research has shown that LNP caused up to 98% of tumor cells to have their genes altered, which considerably slowed down the growth of the tumor cells. Most LNPs administered intravenously become lodged in the hepatocytes and, thereby, ineffectively absorbed by cancer cells. LNPs were altered for targeting tumor using anti-human EGFR antibodies in order to solve this issue. In vivo data demonstrated approximately 70% of PLK1 gene editing following a single dose of single guide PLK1-cLNPs in glioblastoma mouse. This gene editing caused an elevation by almost half in median survival and a one-third elevation in overall survival in glioblastoma mouse. Likewise, Tang et al. [143] developed targeted mRNA delivery using NPs by altering the target molecule phenylboronic acid. These NPs were able to specifically identify and target cancer cells that overexpressed sialic acid (SA), enhancing the precision of the delivery system. They created cell-selective mRNA delivery NPs that could identify cancer cells that overexpressed SA. According to the findings, cancer cells had 300 times more targeted NP transfection than noncancerous cells. Moreover, an efficient gene editing rate of 18.7% and robust inhibition of HeLa cell growth were achieved by using NPs to deliver Cas9 mRNA/sgHPV18E6 to tumor cell lines. Besides using tumor-specific ligands in enhancing the delivery of CRISPR-Cas9 gene editing tools to tumors, the Siegwart group has explored a multidimensional delivery approach. Their research demonstrated that targeted mRNA expression in animal lung, spleen, or liver could be accomplished by incorporating an additional selective organ targeting (SORT) lipid into the LNP formulation, thereby improving the precision and effectiveness of the delivery system [144]. Zhang et al. [145] designed a multiplexed dendrimer-based LNP system for co-delivery of focal adhesion kinase (FAK) siRNA, Cas9 mRNA, and sgRNA to target tumors. This approach aims to achieve a dual effect: modulating tumor stiffness through FAK knockdown and upsetting PD-L1 expression via CRISPR-Cas9-mediated gene editing [145]. Additionally, immune cell-targeting gene editing has been studied as a cancer treatment. Cas mRNA-based therapy has shown an ability to reprogram immunocytes in contrast to the popular immune cell engineering techniques of electroporation and viral transduction [55,146]. The successful application of CRISPR-Cas9 technology in a 2016 Chinese clinical trial to eliminate PD-1 expression in T cells spurred a surge of investigations into its use for CAR-T cell engineering. This approach has rapidly translated into clinical trials targeting various malignancies. For instance, CTX-110, an allogeneic CAR-T therapy leveraging CRISPR-Cas9 to target CD19, is currently undergoing a phase I trial (NCT04035434) for CD19^+^ B-cell malignancies. Similarly, CTX-120, another allogeneic CAR T therapy against BCMA for multiple myeloma, entered a phase I trial (NCT04244656) in the start of 2020. Furthermore, CTX-130, a CD70-directed T-cell therapy utilizing CRISPR-Cas9-modified allogeneic T lymphocytes, is being evaluated in a phase I trial (NCT04502446) for diffuse large B-cell lymphoma and relapsed/refractory T-cell malignancies.

In short, CRISPR-Cas9 shows immense promise for precise gene editing with applications in gene therapy and cancer therapy; however, efficient delivery of CRISPR-Cas9 mRNA to target cells remains a hurdle. Further research is warranted to develop advanced NP platforms with improved biodistribution and controlled release mechanisms to ensure targeted delivery of CRISPR-Cas9 mRNA to specific cells within the body.

## Conclusions and Future Directions

Despite notable progress in reducing overall mortality rates, cancer therapies still face challenges like efficient delivery, immunogenicity, stability, degradation, tumor heterogeneity, and off-target toxicity, thereby warranting investigations into the development of targeted therapeutics. mRNA has emerged as a promising therapeutic option due to its design flexibility, scalability for industrial production, and ability to achieve high cell transfection rates through nanodots and organ selectivity via multidimensional screening.

The mRNA cancer vaccines are recognized for their safety, efficiency, and versatility in generating cancer-specific immune responses, which currently offers promising prospects for cancer immunotherapy. In clinical settings, mRNA vaccines are progressing to utilize personalized approaches like DC-based vaccinations and NP formulations that demonstrates efficacy in not only enhancing antitumor responses but also investigating potential synergies with immunotherapy and chemotherapy. In this case, more investigations for optimal timing and combinations of cancer nanovaccines with immune checkpoint inhibitors like anti-PD-1 therapy are warranted in pursuit of enhanced treatment efficacy and suppressed tumor recurrence. Moreover, we emphasize exploring biomarkers that predict patient subgroups most likely to benefit from specific combinations such as nivolumab/chemotherapy, which would not only aid in personalized treatment strategies but also help in improving clinical outcomes. Lastly, continued research into mRNA vaccines and personalized DC-based vaccines are crucial to focus enhancing immunogenicity and efficacy across solid tumors, such as, melanoma, lung cancer, and CRC.

The success of NP-mediated mRNA delivery hinges on optimizing LNPs for immune cell targeting, activating STING pathways with cyclic lipid LNPs, and leveraging nanocarriers for precise drug delivery and enhanced tumor inhibition. However, future efforts could focus on refining NP designs to improve targeting specificity and efficacy in delivering mRNA vaccines to specific immune cell populations to enhance immune response activation and antitumor effects. Moreover, investigating the combination of NPs with immunomodulators or adjuvants to synergistically enhance STING pathway could amplify antitumor immune responses induced by mRNA vaccines.

In mRNA-based antibody therapies, one critical challenge is ensuring the sustained presence and stability of mRNA-encoded antibodies at therapeutic levels within target tissues. Future research could focus on developing novel mRNA modifications or encapsulation strategies within NPs that enhance mRNA stability and prolong protein expression. This may include exploring modified nucleotides or protective coatings for LNPs that shield mRNA from degradation. Another area needing improvement is the specificity and efficacy of mRNA-based antibodies in targeting tumor antigens while minimizing off-target effects. As we aforementioned, current approaches often rely on delivering mRNA-encoded antibodies broadly, which may not achieve optimal localization to tumor cells or TME. For this, future solutions could involve engineering mRNA sequences to include tumor-specific targeting motifs or enhancing the design of LNPs for improved tissue penetration and cellular uptake.

In case of mRNA-based costimulatory agents, optimization of mRNA delivery to ensure sustained and effective expression of costimulatory molecules in T cells within the TME is still needed to be sorted out. For this challenge, there is a need to develop next-generation NP delivery systems that enhance mRNA stability, cellular uptake, and intracellular trafficking specifically in T cells via involving the use of LNPs engineered to target T cells or biodegradable polymers that protect mRNA from degradation and facilitate controlled release within the TME. Another issue is related to immunosuppressive mechanisms within the TME that limit the effectiveness of costimulatory agents delivered via mRNA. Therefore, we advise to combine mRNA-based costimulatory agents with ICIs to synergistically enhance antitumor immune responses. This approach could involve co-formulating ICIs with mRNA-loaded NPs to achieve localized and sustained delivery. Furthermore, integrating strategies to modulate cytokine profiles or induce specific immune cell subsets, such as regulatory T-cell inhibition or M2 macrophage repolarization, could further potentiate the therapeutic efficacy of mRNA-based costimulatory agents in cancer treatment.

As discussed in our review, mRNA-based cytokine therapies show promise. However, their efficacy can be limited by the immunosuppressive TME, which may counteract the immune-stimulatory effects of cytokines. Thus, investigating combination therapies that integrate mRNA-based cytokines with other immunomodulatory agents, such as immune checkpoint inhibitors (e.g., PD-1/PD-L1 blockers), STING agonists, or bispecific antibodies targeting immunosuppressive factors, could synergistically enhance the activation and persistence of antitumor immune responses.

mRNA-based protein replacement therapy is facing a major hurdle in delivering mRNA efficiently to target cells and subcellular compartments. Notably, ensuring that mRNA reaches the correct intracellular locations to produce therapeutic proteins effectively is critical. To tackle this problem, research should focus on developing advanced nanocarriers such as LNPs, with specific surface modifications, for instance, targeting ligands (e.g., antibodies and peptides), to facilitate targeted delivery to specific cell types and subcellular compartments. Moreover, incorporating CPPs into NP formulations can enhance cellular uptake and endosomal escape in order to ensure that the mRNA reaches the cytoplasm where protein translation occurs.

We have already reviewed and addressed efficient delivery mRNA to T cells to engineer CAR-T cells while minimizing toxicity and maintaining cell viability [19,120]. However, we here suggest to develop and optimize nonviral NP delivery systems specifically tailored for T cells. A systematic optimization strategy, like an orthogonal DoE, could be employed to identify the most effective combination of lipid components. This includes ionizable lipids, PEGylated lipids, and cholesterol, with the goal of maximizing mRNA delivery efficiency and CAR expression. Moreover, to overcome high costs and complexity of ex vivo CAR T-cell manufacturing, research should focus on developing injectable LNP systems capable of delivering mRNA encoding CAR constructs directly to circulating T cells in vivo via creating LNP formulations that can efficiently target and transfect T cells within the body.

Lastly, during our review, we observe a critical challenge for mRNA-based gene editing via CRISPR-Cas9, which is minimizing off-target effects while maximizing precision in editing the desired genomic sites. Off-target effects can lead to unintended genetic modifications, causing potential safety concerns and reducing the efficiency of therapeutic interventions. For this purpose, we suggest LNP modifications as discussed earlier in this section, and using SORT LNP formulations as investigated in studies by Rosenblum et al. and Cheng et al. [142,144].

To sum up, the landscape of cancer treatment is undergoing a transformative phase with the advent of mRNA-based therapeutic applications. The remarkable progress made in mRNA vaccines, nanotherapies, gene editing, and cell-based therapies opens exciting avenues for developing highly efficacious and personalized cancer therapy. As research continues to unfold, the future holds great promise for mRNA-based approaches to play a pivotal role in the ongoing fight against cancer and other genetic disorders.
