# Supplementary material for: Current Trends in Messenger RNA Technology for Cancer Therapeutics
Source: Biomater Res. 2025 Apr 9;29:0178. doi: 10.34133/bmr.0178 (PMC11978394; doi:10.34133/bmr.0178)
Supplement: Supplementary 1 — Fig. S1 [file bmr.0178.f1.docx]

**Supplementary Material**

**Current trends in mRNA technology for cancer therapeutics**

Ali Afzal^1,#^, Muddasir Hassan Abbasi^2,#,*^, Shaaf Ahmad^3,#^, Nadeem Sheikh^4,*^, Muhammad Babar Khawar^1,*­^

^1^Applied Molecular Biology and Biomedicine Lab, Department of Zoology, University of Narowal, Narowal, Pakistan.

^2^Department of Zoology, University of Okara, Okara, Pakistan.

^3^King Edward Medical University/Mayo Hospital, Hospital Road, Lahore, Punjab 54000-Pakistan.

^4^Cell & Molecular Biology Lab, Institute of Zoology, University of the Punjab, Lahore, Pakistan.

**#These authors contributed equally to this study.**

**Correspondence should be addressed to:** [babarkhawar@yahoo.com](mailto:babarkhawar@yahoo.com); [dr.mudassir@uo.edu.pk](mailto:dr.mudassir@uo.edu.pk); nadeem.zool@pu.edu.pk

| **A.**  **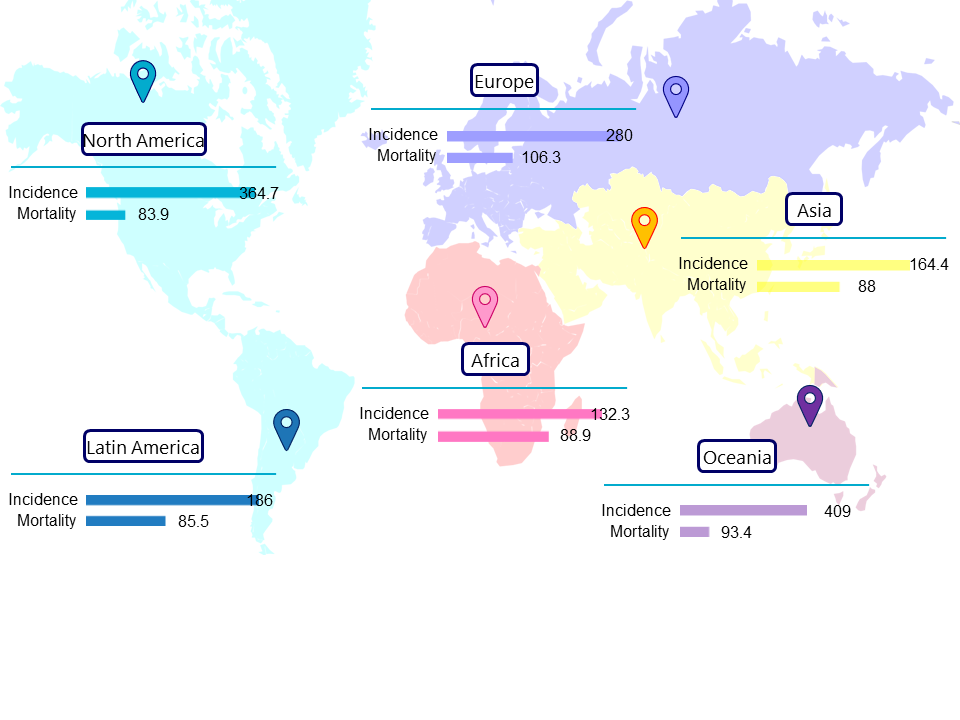** | |
| --- | --- |
| **B.** | |
| **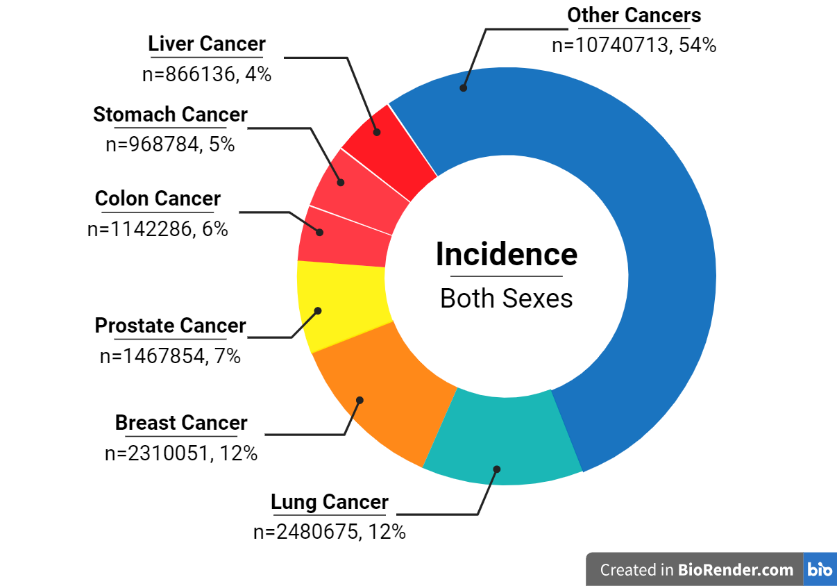** | **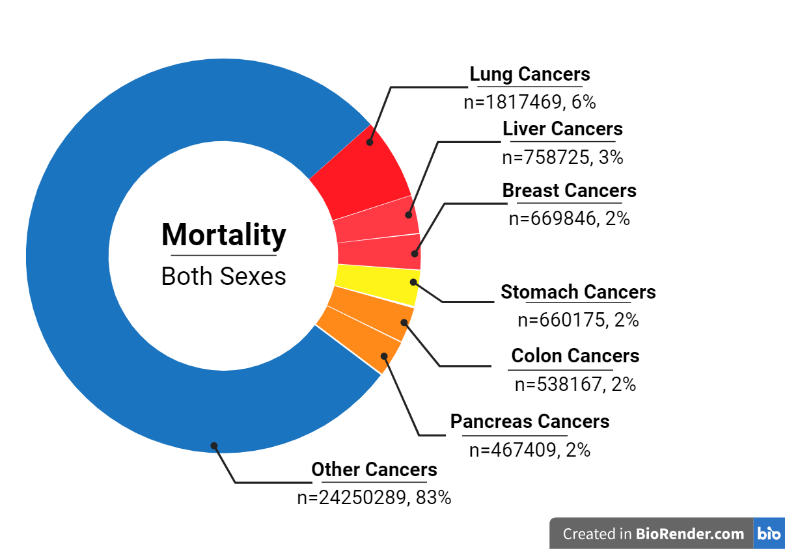** |
| **C.**  **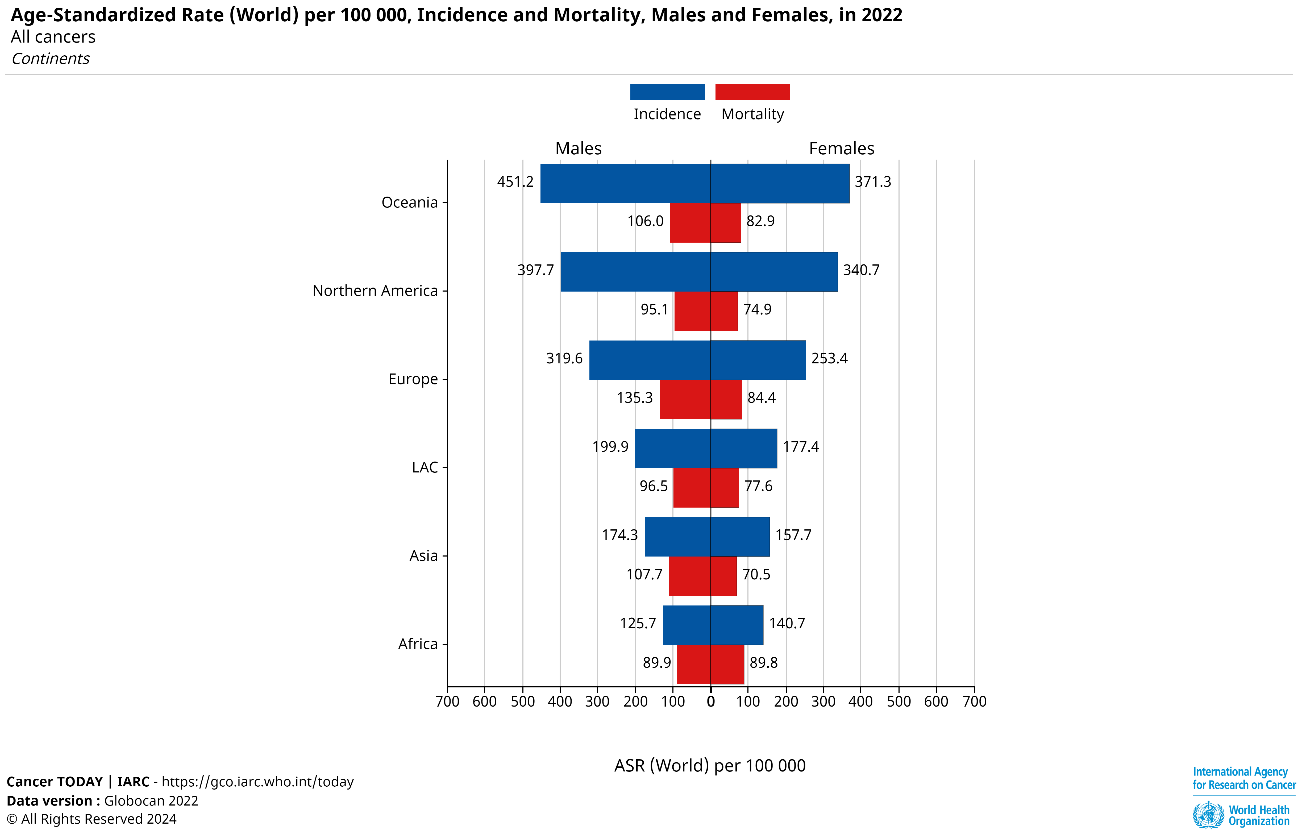** | |

**Figure S1: Global statistics of Cancer in 2022.** A. The world map illustrates the incidence and mortality rates in both sexes for the year 2022. Oceania has recorded the highest incidence, whereas Europe has the highest mortality rates. Africa exhibits the lowest incidence, while America has the lowest mortality, possibly due to advanced healthcare facilities. B. Cancers with the highest incidence and mortality are highlighted, with lung cancer standing out as having the highest rates in both sexes. C. The bar chart indicates a higher incidence and mortality in males compared to females. (Data used in this figure are sourced from GLOBOCAN Cancer Today, 2022).
